# Comparison of artificial intelligence–enhanced electrocardiography approaches for the prediction of time to mortality using electrocardiogram images: reply

**DOI:** 10.1093/ehjdh/ztaf052

**Published:** 2025-05-15

**Authors:** Partha Pratim Ray

**Affiliations:** Department of Computer Applications, Sikkim University, Gangtok, PO Tadong 737102, Sikkim, India

The article by Sau *et al*.^1^ delivers the first rigorous comparison of three deep-learning strategies for estimating an individual’s time to death from an electrocardiogram: a one-dimensional convolutional neural network (1D CNN) trained on natively digital waveforms, a 1D CNN trained on signals digitized from Electrocardiogram (ECG) images, and a two-dimensional CNN (2D CNN) that ingests the image of the tracing itself. This design addresses a central practical obstacle—most healthcare facilities outside major referral centres still archive ECGs as paper or static PDF print-outs and cannot export Extensible Markup Language (XML) signals. By demonstrating that both image-based pipelines match the accuracy of native-signal models, the authors offer a realistic path towards democratizing artificial intelligence (AI)-assisted cardiovascular risk stratification.

Three technical observations in the study deserve emphasis because they anchor any discussion of implementation. First, the digitization pipeline proved highly faithful: across 1 163 401 ECGs in the Beth Israel Deaconess Medical Center derivation set, the median Pearson correlation between each digitized lead and its XML reference was 0.98, with a mean absolute error of only 0.014 mV. Such fidelity confirms that the prognostic features exploited by signal-based networks survive conversion from image to waveform. Second, when the authors retrained the native-signal network on asynchronous 2.5-s segments—mirroring the layout of standard print-outs—the resulting model maintained its concordance index (≈0.78) when applied to digitized data, whereas a network trained on 10-s synchronous inputs lost discrimination (*C*-index 0.737). The implication is clear: legacy 1D CNNs can be translated to image archives provided they are trained, or re-trained, on asynchronous inputs that reflect real-world print formats. Third, the EfficientNet-derived 2D CNN achieved a *C*-index of 0.78 at 310 × 868 pixels and retained values above 0.75 even when the image was down-sampled to 27 × 76 pixels. External validation confirmed robustness: in the CODE cohort, the image network performed slightly better than the signal network (0.767 vs. 0.762, *P* < 0.0001), while in the SaMi-Trop Chagas cardiomyopathy cohort, their performances were statistically indistinguishable (0.747 vs. 0.762, *P* = 0.28). These findings open two complementary routes for institutions with heterogeneous infrastructure: a digitized-signal pipeline that can reuse existing asynchronous 1D models and a ‘plug-and-play’ image pipeline that functions even on low-resolution scans where digitization may fail.

Because the study’s comparative design already encompasses both solutions, it also clarifies how equitable access might be achieved without imposing prohibitive capital expenses. Rural clinics and community hospitals that lack modern ECG carts can immediately deploy the 2D CNN model using inexpensive flatbed scanners; centres with modest processing capacity can install the open-source digitizer and apply an asynchronous 1D CNN without changing their acquisition hardware. Either pathway avoids cost-intensive upgrades to networked XML capable machines, thereby narrowing the technology gap that contributes to cardiovascular outcome disparities across socioeconomic and geographic lines.

Equitable deployment nevertheless requires systematic fairness testing at the subgroup level. Performance should be reported for age, sex, ethnic background, and comorbidity strata and for each acquisition format, because hidden stratification can erode accuracy in underrepresented groups.^2,3^ Corrective measures—such as targeted data augmentation or domain adaptive fine-tuning—must precede clinical rollout whenever material disparities emerge.

Parallel ethical considerations arise because time-to-death estimation touches on patient autonomy, psychological well-being, and potential downstream effects on insurance or resource allocation. Transparent governance frameworks therefore need to define minimum discrimination and calibration targets, establish uniform language for including risk trajectories in electronic health record reports, schedule periodic audits for model drift, and mandate encryption and access controls for both images and derived signals.^4,5^ Although ECG acquisition is commonplace, its secondary use for long-term prognostication warrants concise consent materials that explain benefits, limitations, and opt-in or opt-out choices, thereby preserving trust and respecting autonomy.^6–8^

Future studies should build directly on the empirical groundwork established by Sau *et al*.:

- Apply saliency methods—integrated gradients for 1D CNNs and Grad-CAM for 2D CNNs—to reveal how features such as QRS width, ST–T morphology, and lead-specific.
- Deviations drive risk estimates, providing clinicians with interpretable cues.
- Design prospective trials to determine whether presenting individualized mortality trajectories alters treatment decisions, referral patterns, or patient outcomes.
- Conduct health economic evaluations to measure the cost-effectiveness of these AIECG pipelines, especially in resource-limited environments.
- Broaden training datasets to include varied paper qualities, printer calibrations, and scanner resolutions, reducing hidden stratification errors and preserving model accuracy across global settings.

Sau *et al*. have therefore supplied more than a technical proof-of-concept: they have mapped a credible strategy for bringing AI-based mortality prediction to every clinic that performs an ECG, regardless of whether the tracing emerges as XML, PDF, or faded thermal paper. By retaining accuracy across formats, by clarifying the conditions under which legacy 1D models can be repurposed, and by demonstrating that low-resolution images suffice, the work removes one of the last infrastructural barriers to equitable AI-ECG deployment. With appropriate fairness auditing, transparent governance, and mindful consent practices, the approaches compared in this study can help translate deep-learning insights into tangible improvements in cardiovascular care across the globe.

## Data Availability

No new data were generated or analysed in support of this research.
